# Supplementary material for: Asynchronous magnetic resonance elastography: Shear wave speed reconstruction using noise correlation of incoherent waves
Source: Magn Reson Med. 2022 Oct 27;89(3):990–1001. doi: 10.1002/mrm.29502 (PMC9792433; doi:10.1002/mrm.29502)
Supplement: Supplementary file 1 — DATA S1 MATLAB code used to generate the numerical simulations of Figure 1 [file MRM-89-990-s002.zip › k-Wave/helpfiles/makeDisc.html]

makeDisc :: Functions (k-Wave)


# makeDisc

Create a binary map of a filled disc within a 2D grid.

## Syntax

```
disc = makeDisc(Nx, Ny, cx, cy, radius)
disc = makeDisc(Nx, Ny, cx, cy, radius, plot_disc)
```

## Description

`makeDisc` creates a binary map of a filled disc within a two-dimensional grid (the disc position is denoted by 1's in the matrix with 0's elsewhere). A single grid point is taken as the disc centre thus the total diameter of the disc will always be an odd number of grid points. As the returned disc has a constant grid point radius, if used within a k-space grid where dx and dy are not equal, the disc will appear oval shaped. If part of the disc overlaps the grid edge, the rest of the disc will wrap to the grid edge on the opposite side. The plot that is displayed after calling `makeDisc(60, 100, 30, 50, 20, true);` is given below.

## Inputs

|  |  |
| --- | --- |
| `Nx, Ny` | size of the 2D grid [grid points] |
| `cx, cy` | centre of the disc [grid points] |
| `radius` | disc radius [grid points] |

## Optional Inputs

|  |  |
| --- | --- |
| `plot_disc` | Boolean controlling whether the disc is plotted using `imagesc` (default = false) |

## Outputs

|  |  |
| --- | --- |
| `disc` | 2D binary map of a filled disc |

## Examples

- Homogenous Propagation Medium
- Using A Binary Sensor Mask
- Heterogeneous Propagation Medium
- Saving Movie Files
- Recording The Particle Velocity
- Defining A Gaussian Sensor Frequency Response
- Comparison Of Modelling Functions
- Setting An Initial Pressure Gradient
- Photoacoustic Waveforms in 1D, 2D and 3D
- Focussed Detector in 2D
- 2D FFT Reconstruction For A Line Sensor
- 2D Time Reversal For A Line Sensor
- Image Reconstruction With Directional Sensors
- Image Reconstruction With Bandlimited Sensors
- Image Reconstruction With Compensation For Acoustic Absorption
- Controlling The Absorbing Boundary Layer

## See Also

`makeCircle`, `makeBall`
